# Medication Experience and Associated Factors in Older Adults with Multiple Chronic Conditions in Rural Henan Province, China: A Single-Center Cross-Sectional Study

**DOI:** 10.3390/healthcare14070965

**Published:** 2026-04-07

**Authors:** Xiaofan Wang, Linlin Su, Xiao Yang, Ruofan Qiao, Jixuan Zheng, Chunhui Zhang, Xian Zhang, Lixia Qu, Beilei Lin

**Affiliations:** School of Nursing and Health, Zhengzhou University, Zhengzhou 450001, China; wangxf202409@163.com (X.W.); sulinlin2023@gs.zzu.edu.cn (L.S.); zzu2024yx@163.com (X.Y.); qrf200302@163.com (R.Q.); zjx15038284278@126.com (J.Z.); qulixia@zzu.edu.cn (L.Q.); linbeilei@zzu.edu.cn (B.L.)

**Keywords:** medication experience, chronic disease, comorbidity, older adults, rural

## Abstract

**Highlights:**

**What are the main findings?**

- The medication experience of older adults with multiple chronic conditions in the rural county of Henan Province, China, was at a moderate level.
- Age, marriage status, source of income, medical insurance schemes, duration of medication use, safe medication knowledge, perceived social support, self-efficacy for chronic disease management, and medication errors were factors significantly associated with patients’ medication experience.

**What are the implications of the main findings?**

- The findings suggest that multidimensional strategies—with emphasis on safe medication knowledge, perceived social support, self-efficacy for chronic disease management, and medication errors—may be beneficial for medication experience in this population.
- When developing medication management plans, healthcare professionals may consider these modifiable factors associated with medication experience to design personalized and structured support strategies, which may contribute to better medication safety and experience.

**Abstract:**

**Objectives:** We aimed to investigate the medication experience of older adults with multiple chronic conditions in rural areas and to analyze its associated factors, so as to provide evidence for developing targeted medication management interventions. **Design:** This was a single-center cross-sectional study. **Methods:** From June to July 2025, a convenience sample of 539 older adults with multiple chronic conditions was recruited from a county hospital in Henan Province, China. The survey utilized a general information questionnaire, the Chinese version of the Medication Experience Scale, the Safe Medication Knowledge Scale, the Perceived Social Support Scale, the Chinese version of the Chronic Disease Self-Efficacy Scale, and the Chinese version of the Medication Errors Scale. **Results:** The medication experience score among rural older adults with multiple chronic conditions was (117.14 ± 17.19). Multivariate hierarchical regression analysis revealed that age, marriage status, source of income, medical insurance schemes, duration of medication use, safe medication knowledge, perceived social support, self-efficacy for chronic disease management, and medication errors were significant factors associated with medication experience (all *p* < 0.05). **Conclusions:** The medication experience among older adults with multiple chronic conditions in our study sample was at a moderate level. Age, marriage status, source of income, medical insurance schemes, duration of medication use, safe medication knowledge, perceived social support, self-efficacy for chronic disease management, and medication errors were associated factors of medication experience for older adults with multiple chronic conditions. Countermeasures should be implemented to improve medication experience in this population.

## 1. Introduction

Over the last few decades, under the influence of various factors such as population aging, lifestyle changes, and environmental pollution, chronic diseases have become the most important global disease burden and the leading cause of disability and death [1]. The World Health Organization defines multiple chronic conditions (MCCs) as the coexistence of two or more chronic conditions in an individual [2]. Studies indicate that the prevalence of MCCs among older adults aged 60 and above in China ranges from 6.4% to 76.5% [3,4]. The presence of multimorbidity significantly complicates chronic disease management, leading to numerous challenges such as polypharmacy, difficulties in medication management, decreased medication adherence, increased treatment burden, impaired quality of life, and elevated mortality risk [5]. These issues pose serious challenges to the health of the entire population and to the construction of Healthy China [6].

The concept of “medication experience” was initially proposed by Shoemaker et al. in 2007 [7], referring to the subjective perceptions and feelings individuals develop through the daily routine of taking medication. This construct encompasses how patients’ medication-related needs are met and is broadly categorized into positive and negative experiences. A positive medication experience refers to patients adapting to daily life with medication, learning and reflecting on medication-related information, and managing side effects, feelings of insecurity, and discomfort arising during the medication process. A negative medication experience refers to patients experiencing negative emotions and non-adherent behaviors due to factors such as low satisfaction with medication, a high burden of treatment, or poor medication practices, ultimately affecting their overall physical and mental health [8,9]. As the concept evolved, in 2020, Hillman et al. [10] described the patient medication experience as “the sum of all events involving drug therapy that a patient encounters throughout their life, encompassing their perceptions and feelings regarding the benefits gained from and the burdens associated with medication therapy.” Research indicates that prioritizing patient medication experience is essential for improving patient outcomes and ensuring medication safety. Positive medication experiences have been shown to improve patient adherence to medication regimens, thereby further promoting patient recovery [10,11]. Conversely, negative medication experiences can not only affect patients’ beliefs about their medication but also lead to numerous medication-related problems, potentially precipitating physiological, psychological, and social functional impairments [12,13].

The complexity of patient responses to medication is well-documented. A retrospective cohort study by Wright et al. [14] demonstrated that an increasing medication regimen exacerbates treatment burden, often resulting in negative medication experiences. Additionally, concerns regarding drug resistance and safety can trigger resistance in patients, resulting in poor medication experiences and ultimately delaying the recovery process [15]. However, a systematic review by Niu et al. [16] revealed that MCCs often cultivate a positive medication experience through active psychological adjustment. Specifically, they maintain an optimistic outlook by focusing on the tangible benefits of treatment, such as improved quality of life and extended time with family, and hold a firm belief that medication is both necessary and effective for preserving their health.

The medication experience of MCCs is shaped by a range of factors. Patients’ knowledge of safe medication use [17], emotional support from family and informational support from the broader social network [18], along with higher self-efficacy [19], have emerged as key factors associated with improved health behaviors and medication adherence [20]. Collectively, these factors motivate patients to maintain a favorable medication status, thereby fostering a positive medication experience. Therefore, focusing on the medication experience of older adults with MCCs provides a new perspective for addressing polypharmacy issues in this vulnerable population. Nevertheless, research on this topic has remained relatively scarce in recent years. This gap is particularly pronounced in rural areas, where older adults with MCCs face greater difficulties in chronic disease management due to multiple disadvantages, including limited accessibility to healthcare services, lower health literacy, and inadequate support for medication management [21]. Among these challenges, issues related to safe and standardized medication use are especially prominent [22]. Therefore, this study aims to investigate medication experience and its associated factors among older adults with MCCs in rural areas. It seeks to provide targeted entry points for improving the medication experience of older adults with MCCs, and to offer scientific evidence for enhancing medication safety, promoting health and well-being, and advancing health equity in this population.

## 2. Methods

### 2.1. Participants

A convenience sampling method was used to recruit older adults with MCCs admitted to a county hospital in Kaifeng, Henan Province, China from June to July 2025 as the study participants. Inclusion Criteria: (1) age ≥ 60 years; (2) were engaged in farming as their primary occupation and had resided continuously in rural areas for at least six months, with rural status defined according to the National Bureau of Statistics’ urban-rural classification standard; (3) diagnosed with at least two chronic conditions (including stroke, coronary heart disease, hypertension, diabetes, chronic bronchitis, etc.) by a secondary-level or higher hospital; (4) provided informed consent and voluntarily agreed to participate in this study. Exclusion Criteria: (1) history of cognitive impairment or mental illness; (2) patients in the acute or terminal phase of illness; (3) currently participating in other research studies.

### 2.2. Sampling

Based on the rule-of-thumb estimation method for sample size in cross-sectional studies, the sample size should be 5 to 10 times the number of variables [23]. Furthermore, to account for potential non-responses, the sample size calculation should be increased by an additional 20%. The formula is N = [(5 − 10) × *n*]/(1 − 0.2). This study encompassed 23 variables; thus, using the formula N = (23 × 5)/0.8, the minimum required sample size was calculated to be 144. In total 539 cases were finally included in this study.

### 2.3. Measures

#### 2.3.1. Demographics

Patient demographic characteristics (including gender, age, educational level, marriage status, etc.) and disease-related information (such as types of illness, duration of illnesses, number of medication, and duration of medication use).

#### 2.3.2. Chinese Version of the Medication Experience Questionnaire for Older Adults with MCCs

Medication experience was assessed using a self-report instrument developed by the research team [24] to assess the medication experience of older adults with MCCs. Initially, dimensions were theoretically grounded in the Strategic Experience Modules framework [25], with the item pool generated from a literature review, qualitative interviews, and the conceptual framework of patients’ lived medication experience [26]. All items were developed in Mandarin for the target population of older adults in China, with attention to clarity and brevity. Content validity was established through a two-round Delphi survey involving 15 experts in medication management and gerontological nursing, yielding a scale-level content validity index (S-CVI) of 0.958 and item-level CVI (I-CVI) ranging from 0.875 to 1.000. A pre-test among 58 community-dwelling older adults confirmed item comprehensibility. Psychometric evaluation was conducted on a formal sample of 586 participants. Exploratory factor analysis extracted five factors explaining 70.53% of the variance, with factor loadings ranging from 0.695 to 0.919. Confirmatory factor analysis demonstrated good model fit(χ^2^/df = 1.150, GFI = 0.902, NFI = 0.927, IFI = 0.990, CFI = 0.990, AGFI = 0.902, RMSEA = 0.021). The scale exhibited robust convergent validity (AVE > 0.5, CR > 0.7 for all dimensions) and discriminant validity (Fornell-Larcker criterion satisfied). Reliability was satisfactory, with an overall Cronbach’s α of 0.922, dimension-specific α ranging from 0.874 to 0.945, split-half reliability of 0.888, and two-week test–retest reliability of 0.886. Completion time averaged 10–20 min. These findings demonstrate that the scale possesses sound reliability, validity, and feasibility for assessing medication experience in this population. The final scale comprises five dimensions—sensory experience, emotional experience, cognitive experience, behavioral experience, and relational experience—with a total of 36 items. Items are rated on a 5-point Likert scale, with total scores ranging from 36 to 180. Scores ≤ 60 are classified as the low-score group, 61–120 as the medium-score group, and ≥ 121 as the high-score group. Higher scores indicate a more positive medication experience. In this study, the Cronbach’s α coefficient for this questionnaire was 0.907.

#### 2.3.3. Medication Safety Knowledge Scale

The Medication Safety Knowledge Scale, originally developed by Ma Xiangqin [27] in 2015, was utilized in this study. This assessment tool consists of 12 items. Each item is rated on a 4-point Likert scale from 0 to 3, with response options ranging from “unaware” (0) to “fully understand” (3). Total scores range from 0 to 36, with higher scores indicating greater medication safety knowledge. Based on predefined cutoff points, scores of 29–36 are classified as “good”, 20–28 as “fair”, and 0–19 as “poor”. The Cronbach’s alpha coefficient for the scale in this study was 0.841.

#### 2.3.4. Perceived Social Support Scale

The Perceived Social Support Scale, originally developed by Zimet et al. [28] in 1987, was subsequently introduced into the Chinese context by Jiang Qianjin [29]. The Chinese version has been validated and demonstrates good reliability and validity for use in the adult population. This scale comprises 12 items assessing support across three dimensions: family, friends, and significant others. Items are rated on a 7-point Likert scale ranging from “strongly disagree” (1 point) to “strongly agree” (7 points). Total scores are proportional to the level of perceived social support, categorized as follows: 12–36 indicates low support, 37–60 represents moderate support, and 61–84 signifies high support. The Cronbach’s alpha coefficient for the scale in this study was 0.916.

#### 2.3.5. Chinese Version of the Self-Efficacy for Managing Chronic Disease Scale

The Self-Efficacy for Managing Chronic Disease Scale (SEMCD) was originally developed by the Stanford University Patient Education Research Center [30]. Lorig et al. [31] simplified the initial 36-item Chronic Disease Self-Efficacy Scale (SEMCD) into a 6-item version, also referred to as the Self-Efficacy for Managing Chronic Disease 6-item Scale (SEMCD6). This scale assesses patients’ confidence in managing specific issues encountered recently, including overcoming fatigue, pain management, emotional management, symptom management, activity management, and medication management. Each item is rated on a 10-point scale ranging from 1 to 10, indicating “not at all confident” to “totally confident.” The total score is calculated as the mean of the six items, with ≤4.0 indicating low level, 4.0–7.9 indicating moderate level, and ≥8.0 indicating high level. This version of the scale has demonstrated sound reliability and validity [32]. The Chinese version was translated and adapted by Zhang Meixia et al. [33], reporting a Cronbach’s α coefficient of 0.934. The Cronbach’s alpha coefficient for the scale in this study was 0.901.

#### 2.3.6. Chinese Version of the Medication Error Questionnaire

The Medication Error Questionnaire is a self-report questionnaire developed by Chinese scholar Zhang Huiling [34] based on the medication error classifications defined by the American Society of Health-System Pharmacists [35], adapted to the context of medication errors among older adults with cardiovascular disease in China. The scale comprises 10 items covering different aspects of medication errors (e.g., failure to review prescriptions, omissions, dosage errors, timing errors, unauthorized drug use, non-adherence to instructions, monitoring lapses, incorrect dosage forms, use of expired medications, and storage errors). Each item includes four response options: “often,” “sometimes,” “occasionally,” and “never,” representing varying degrees of medication errors. A 4-point Likert scale is employed, with scores ranging from 1 to 4 points corresponding to “often” to “never,” and total scores ranging from 10 to 40. Higher scores indicate a lower incidence of medication errors. The Cronbach’s alpha coefficient for the scale in this study was 0.817.

### 2.4. Data Collection

Data collection was conducted by three researchers (XFW, LLS, XY), all of whom had completed standardized training on questionnaire distribution and administration procedures. After obtaining consent from the department heads, paper-based questionnaires were administered face-to-face. Prior to the survey, the purpose, significance, participation methods, and confidentiality principles of the study were thoroughly explained to the patients. Following informed consent, trained investigators administered the questionnaires in a face-to-face, question-and-answer format. To ensure consistency, standardized instructions were used throughout, and any items that participants found difficult to understand were explained in a uniform manner, thereby maintaining consistent interpretation across all respondents. Upon completion, the questionnaires were immediately reviewed, and any missing items were supplemented on-site to ensure data integrity. Within 24 h of collection, the data were double-checked and entered by two researchers. A total of 551 questionnaires were distributed in this study. After excluding 12 invalid responses, 539 valid questionnaires were collected, yielding a valid response rate of 97.82%.

### 2.5. Statistical Analysis

We conducted all data entry and statistical analysis using IBM SPSS^®^ Statistics version 25.0 (SPSS Inc., Chicago, IL, USA). Normality was assessed using Q-Q plots and skewness and kurtosis values (±2), which indicated that the core variables were approximately normally distributed. Normally distributed continuous variables were described using mean ± standard deviation, while categorical variables were described using frequency and proportion. To identify potential factors associated with medication experience, we first conducted univariate analyses. For categorical variables, differences in medication experience scores were examined using independent-sample t-tests for binary variables (e.g., gender, age, types of illness) and one-way analysis of variance (ANOVA) for variables with more than two categories (e.g., educational level, marriage status, monthly household income per capita, sources of income, medical insurance schemes, duration of illness, number of medications, duration of medication use). Correlations between variables were analyzed using Pearson correlation analysis. Multiple linear regression analysis was employed to identify factors associated with medication experience in this population. The test level α = 0.05, *p* < 0.05 indicates that the difference is statistically significant.

### 2.6. Ethical Considerations

This study adheres to the Declaration of Helsinki and was approved by the Ethics Committee of Zhengzhou University (Approval No. ZZUIRB2025-56) on 17 January 2025. All participants were informed of the purpose and content of the study. Personal information obtained in this study was anonymized and kept strictly confidential. Written informed consent was obtained from participants. Participants were informed that they could withdraw from the study at any time.

## 3. Results

### 3.1. Common Method Bias

As the data for all major variables were collected through self-report questionnaires in a single survey, there is a potential risk of common method bias. To enhance rigor, we conducted Harman’s single-factor test using unrotated exploratory factor analysis. The results revealed 15 factors with eigenvalues greater than 1, with the first factor accounting for 21.5% of the total variance, which is below the recommended threshold of 40% [36]. These findings suggest that common method bias is unlikely to be a serious concern in this study.

### 3.2. General Characteristics of Rural Older Adults with MCCs

Among the 539 participants, males accounted for 50.65% and females for 49.35%. The majority were aged 60 to 75 years (73.7%), 65.3% were prescribed three or more types of medications, and 81.6% had been on medication for more than three years. Additional demographic characteristics are presented in Table 1.

### 3.3. Scores for Medication Experience, Safe Medication Knowledge, Perceived Social Support, Self-Efficacy for Chronic Disease Management, and Medication Errors in Rural Older Adults with MCCs

In this study, the scores of medication experience, safe medication knowledge, perceived social support, self-efficacy for chronic disease management, and medication errors among rural older adults with MCCs were (117.14 ± 17.19), (11.99 ± 5.84), (58.08 ± 10.37), (5.97 ± 1.58), and (27.97 ± 5.31), respectively. Specific scores of each scale are shown in Table 2.

### 3.4. Univariate Analysis of Medication Experience in Rural Older Adults with MCCs

Univariate analysis revealed statistically significant differences (*p* < 0.05) in medication experience scores among older adults with MCCs based on gender, age, educational level, monthly household income per capita, source of income, medical insurance schemes, and duration of illness (see Table 1).

### 3.5. Correlation Analysis of Medication Experience with Medication Safety Knowledge, Social Support, Self-Efficacy for Chronic Disease Management, and Medication Errors Among Rural Older Adults with MCCs

Pearson correlation analysis revealed that medication experience among rural older adults with MCCs was positively correlated with safe medication knowledge (*r* = 0.590), perceived social support (*r* = 0.565), self-efficacy for chronic disease management (*r* = 0.546), and medication errors (*r* = 0.415), all *p* < 0.001.

### 3.6. Multiple Linear Regression Analysis of Medication Experience Among Rural Older Adults with MCCs

Hierarchical multiple regression analysis was conducted to examine factors associated with medication experience among rural older adults with MCCs. The assignment of independent variables was shown in Table 3.

In Model 1, demographic characteristics (gender, age, marriage status) were entered. The results showed that gender (β = −0.104, *p* = 0.015) and age (β = −0.104, *p* = 0.017) were significantly associated with medication experience. This model explained 2.7% of the total variance in medication experience (F = 4.959, *p* = 0.002).

In Model 2, socioeconomic indicators (educational level, monthly household income per capita, sources of income) were added. The results showed that educational level (β = 0.174, *p* < 0.001) and monthly household income per capita (β = 0.190, *p* < 0.001) were marginally significant. Gender and age lost significance after socioeconomic indicators were added. The model explained 15.0% of the total variance, with a ΔR^2^ of 12.3% (ΔF = 15.315, *p* < 0.001).

In Model 3, medical insurance schemes were further included. The results indicated that educational level (β = 0.159, *p* < 0.001), monthly household income per capita (β = 0.175, *p* < 0.001) and other insurance (β = −0.260, *p* < 0.001) were significantly associated with medication experience. This model accounted for 22.1% of the variance, with a ΔR^2^ of 7.1% (ΔF = 24.167, *p* < 0.001).

In Model 4, disease-related characteristics (types of illness, duration of illness, number of medications, and duration of medication use) were added. Educational level (β = 0.155, *p* < 0.001), monthly household income per capita (β = 0.181, *p* < 0.001) and other insurance (β = −0.254, *p* < 0.001) remained significant. The model explained 22.8% of the variance, with a ΔR^2^ of only 0.7% (ΔF = 1.108, *p* = 0.352), which was not statistically significant.

In Model 5, psychosocial factors (medication safety knowledge, perceived social support, and self-efficacy for chronic disease management) were entered. Age (β = −0.070, *p* = 0.019), marriage status (β = 0.094, *p* = 0.002), other income source (β = 0.107, *p* = 0.002), other insurance (β = −0.137, *p* < 0.001), medication safety knowledge (β = 0.355, *p* < 0.001), perceived social support (β = 0.289, *p* < 0.001), and self-efficacy for chronic disease management (β = 0.254, *p* < 0.001) were all significantly associated with medication experience. Educational level and monthly household income per capita lost significance, indicating that their effects were fully mediated by psychosocial factors. The model explained 59.2% of the variance, representing a substantial ΔR^2^ of 36.4% (ΔF = 155.166, *p* < 0.001).

In the final Model 6, medication errors were added. Age (β = −0.090, *p* = 0.002), marriage status (β = 0.098, *p* < 0.001), other income source (β = 0.132, *p* < 0.001), other insurance (β = −0.125, *p* < 0.001), duration of medication use (β = 0.108, *p* = 0.034), medication safety knowledge (β = 0.291, *p* < 0.001), perceived social support (β = 0.294, *p* < 0.001), self-efficacy for chronic disease management (β = 0.226, *p* < 0.001), and medication errors (β = 0.177, *p* < 0.001) remained significant predictors of medication experience. The full model accounted for 61.5% of the total variance, with a ΔR^2^ of 2.3% (ΔF = 30.772, *p* < 0.001). See Table 4.

Partial regression plots showed linear relationships between independent variables and medication experience. The plot of standardized residuals against standardized predicted values exhibited a random distribution, suggesting homoscedasticity. Histograms and P-P plots of standardized residuals approximated a normal distribution. The Durbin-Watson statistic was 1.787, indicating independence of residuals. Variance inflation factors (VIF) for all independent variables ranged from 1.001 to 3.496, well below the recommended threshold of 10, indicating no serious multicollinearity [37]. These results confirm that all assumptions for linear regression were adequately met, supporting the reliability of the findings.

## 4. Discussion

In this study, the total medication experience score of rural older adults with MCCs was (117.14 ± 17.19), indicating a moderate level. This result was higher than that reported by Niu et al. [24] among older adults with MCCs, a discrepancy that may be attributed to the fact that participants in our study were recruited from a county-level hospital, likely representing patients with relatively stable disease conditions and a lower medication burden. Analysis of dimension scoring rates revealed that the behavioral experience dimension had the lowest score. This finding suggests significant difficulties in the accuracy of daily medication execution, adherence, and medication self-management among these patients [38]. The reasons for this may be multifaceted. Firstly, over 80% of the patients in this study had an educational attainment of junior high school or below, and all were from rural areas. This low educational level contributes to insufficient medication literacy, manifesting as potential difficulties in interpreting professional information such as drug instructions and medical prescriptions, which directly undermines their ability to accurately execute medication regimens and leads to poor medication adherence [39]. Secondly, the empty-nest phenomenon in rural family structures has weakened the traditional function of family supervision and support. Research indicates that sustained care and support from family and friends play a significant role in improving patients’ medication experience, thereby reinforcing their motivation to maintain good medication habits [40]; however, rural older adults living alone precisely lack this external support. At the healthcare level, rural areas face challenges such as weak primary care digital infrastructure, a shortage of pharmaceutical care professionals, and fragmented health management, resulting in severely limited accessibility and convenience of professional medication guidance [41]. Therefore, it is suggested that healthcare providers explore the potential of a dual-track intervention model for older adults with MCCs, characterized by a dual-track approach that combines medication behavior observation led by family contract doctors with peer support among neighbors. Specifically, leveraging family doctor contract services, in-home medication behavior observation—such as “watching the patient take a dose”—could be considered as a low-cost strategy for behavioral correction and precise guidance, offering a form of visualized behavioral intervention. Similarly, activating rural social capital by cultivating healthy young-old adults to form neighborhood medication support groups might be considered to address daily reminders, dialect explanations, and emotional companionship, potentially helping to fill the gap left by absent family supervision. Through these measures, medication behavior challenges faced by rural older adults with MCCs may be systematically addressed at the execution level, contributing to an enhanced medication experience in this population.

Several sociodemographic characteristics—including age, marriage status, sources of income, and medical insurance schemes—were associated with medication experience among rural older adults with MCCs. Consistent with the findings of Vaineri et al. [42] and Niu et al. [24], younger age was associated with a more positive medication experience, likely attributable to greater medication management capacity and lower treatment burden. In contrast, older patients tended to report poorer medication experience, largely due to physical decline and memory impairment. Patients with other marital statuses (including unmarried, widowed, etc.) showed better medication experience than married patients, which is in contrast to previous studies reporting that married patients exhibited better medication experience [18]. The discrepancy may be attributed to differences in study populations. Krska et al. [18] included participants across a broad age range, whereas our study focused exclusively on older adults. Among younger adults, spousal support may enhance medication experience through active supervision. In older adults, however, spousal support may be limited due to the spouse’s own aging or declining health. Consequently, older adults with other marital statuses may develop stronger self-management skills and rely more on other family members or community healthcare services, contributing to a better medication experience. Regarding economic factors, patients in receipt of a pension, covered by Urban Employee Basic Medical Insurance, or enrolled in Urban and Rural Resident Basic Medical Insurance reported significantly better medication experience than those without such coverage, aligning with the findings of Burcu et al. [43] and Liu et al. [44]. Stable financial resources and medical insurance coverage alleviate economic burden, thereby enhancing patients’ cooperation with and adherence to pharmacotherapy. These findings carry important clinical implications. Particular attention should be directed toward older adults advanced age, limited financial resources, and less comprehensive medical insurance coverage, for whom alternative support systems—such as home visits and neighborhood mutual assistance—may be beneficial. Furthermore, simplifying medication regimens, using easily identifiable drug packaging, and proactively assessing patients’ economic circumstances to optimize prescription cost-effectiveness—for instance, by prioritizing medications included in insurance formularies—may contribute to a comprehensive improvement in patients’ medication experience.

According to the study’s findings, the duration of medication use was associated with medication experience among rural older adults with MCCs. The results indicated that a longer duration of medication use was associated with a lower level of medication experience. This finding may be attributed to the progressive decline in physiological function and cognitive capacity associated with aging [45], which renders older patients susceptible not only to the cumulative burdens of time, financial cost, and physical exertion associated with long-term pharmacotherapy, but also to an elevated risk of drug–drug interactions and adverse reactions resulting from polypharmacy [46]. The convergence of these factors ultimately exerts a negative impact on patients’ medication experience. Therefore, healthcare providers should pay particular attention to older patients with prolonged medication duration, routinely assess their medication burden and cognitive function, and implement timely simplification of medication regimens alongside targeted guidance, thereby contributing to an improved medication experience in this vulnerable population.

This study reveal that patients’ safe medication knowledge was associated with their medication experience. Patients with a higher level of safe medication knowledge reported better medication experiences, a finding consistent with the research conducted by Sun et al. [47]. The reason may be that patients with greater safe medication knowledge are better able to accurately understand medical instructions and effectively manage their own medication regimens [48], thereby fostering more positive cognitive and behavioral experiences during the medication process. It is noteworthy, however, that some rural older adults struggle to translate medication knowledge into consistent and safe medication habits, which may be attributed to several factors. First, the older adult population exhibits significant declines in executive function [45], manifesting as difficulties in accurately remembering multi-medication dosing schedules and confusing drug names. Second, patients with comorbidities often face polypharmacy regimens [49], involving multiple medications with varying frequencies, which may further increase cognitive load and the difficulty of behavioral execution. Third, rural areas are constrained by weak primary care digital infrastructure, a shortage of pharmaceutical care professionals, and fragmented health management, resulting in insufficient accessibility and convenience of medication guidance [41], which further exacerbates the medication burden on older adult patients. Therefore, it is suggested that healthcare providers consider developing intuitive and easy-to-use medication guidance tools for older adults with MCCs, such as medication flowcharts, daily pill organizers, and brief instructional videos, to help optimize delivery of complex medication information. Building on this foundation, integrating one-on-one medication education and family involvement could be explored to enhance patients’ ability to correctly understand and apply health information, potentially contributing to a virtuous cycle of safe medication use—from identification and understanding to adherence to medical orders—which may ultimately support health improvements and enhance patients’ medication experience.

Our study results reveal that the level of perceived social support was associated with medication experience among rural older adults with MCCs. Higher levels of perceived social support were associated with more positive medication experiences, which is consistent with the findings of KRSKA et al. [50] and Shen et al. [51]. On one hand, patients with higher perceived social support can compensate for declines in self-management ability due to physiological aging through instrumental support such as medication reminders from family members and accompaniment to medical visits, thereby reducing risks like missed or incorrect doses and improving medication accuracy and safety [52]. On the other hand, sustained emotional support also helps enhance patients’ intrinsic motivation, encouraging them to maintain standardized medication behaviors, improve medication adherence, and ultimately foster a more positive medication experience [53]. Therefore, it is suggested that primary healthcare providers consider exploring the establishment of a collaborative support network linking county hospitals, village doctors, and families for patients with weak social support systems. Encouraging family involvement in medication management, along with diversified approaches such as regular broadcast reminders in villages and patient peer support groups, could be considered to help build a sustainable and accessible social support system, potentially contributing to systematically enhancing patients’ medication experience.

The results of this study indicated that patients’ self-efficacy for chronic disease management was associated with their medication experience. Higher levels of self-efficacy were associated with more positive medication experience among rural older adults with MCCs, which is consistent with the findings of Tang et al. [54]. Self-efficacy reflects an individual’s confidence in their ability to perform specific behaviors and achieve goals. Patients with higher self-efficacy are more confident in managing their diseases and adhering to medication regimens, enabling them to proactively seek solutions and demonstrate better adaptability when facing medication-related challenges [55]. This confidence also helps patients maintain a positive coping attitude, reduce feelings of helplessness and avoidance, and thereby sustain stable medication adherence and emotional experience. Conversely, patients with lower self-efficacy in chronic disease management are more prone to feelings of helplessness and avoidance due to lack of confidence, and may resort to inappropriate coping strategies such as self-discontinuation or irregular medication use when encountering difficulties [56], thereby lowering their medication experience. Therefore, for patients with lower self-efficacy, it is suggested that healthcare professionals consider focusing on building their confidence and empowering their behaviors. This could be explored through sharing successful medication management cases, setting phased goals, and other methods aimed at helping patients accumulate successful experiences, gradually build medication confidence, while providing personalized guidance to enhance their problem-solving abilities, potentially contributing to improving their overall medication experience.

The findings reveal that medication errors (with higher scores indicating fewer errors) were positively associated with medication experience. In other words, a lower incidence of medication errors was associated with a higher level of medication experience among rural older adults with MCCs, which is consistent with the findings of McCahon et al. [57]. A lower incidence of medication errors indicates that patients adhere to standardized medication practices and demonstrate good adherence, enabling them to accurately execute prescribed regimens and thereby gain a greater sense of safety and control during the medication process [26]. Patients with a higher incidence of medication errors demonstrate poorer medication adherence, exposing them to greater medication safety risks. This situation can easily lead to doubt regarding their treatment regimen, subsequently inducing anxiety, frustration, or even resistance, ultimately negatively impacting disease management and diminishing their medication experience [57,58]. Furthermore, factors such as a lack of accurate and comprehensive medication guidance, prescription conflicts arising from multiple information sources, and duplicate orders are significant contributors to medication errors among patients with chronic diseases [59]. Among these various influencing factors, medication adherence, as a critical link in reducing medication errors, warrants particular attention. Existing research has shown that the state of the psychological contract between patients and pharmacists is positively correlated with medication adherence [60]. When pharmacists demonstrate professional competence and fulfill their responsibilities for service and empathetic care, it can effectively motivate patients to follow medication guidance, thereby positively influencing adherence [61]. Therefore, for patients with a higher incidence of medication errors, it is suggested that primary healthcare professionals consider assisting them in identifying the causes of these errors and helping to enhance their awareness of medication risks and their ability to proactively seek help. Building on this foundation, exploring the development of a dynamic intervention mechanism based on the psychological contract—by strengthening the trusting interaction and commitment between pharmacists and patients—might be considered to enhance patients’ sense of participation and security during the medication process, potentially contributing to optimizing their overall medication experience.

## 5. Limitations

Several limitations should be noted for this study. First, this study employed convenience sampling and was conducted exclusively at a county-level hospital in Henan Province, China. Although the findings provide reference value for studies under similar economic and cultural conditions, whether the conclusions can represent rural older adults with MCCs in other regions warrants further investigation. Future studies should conduct multicentre surveys using random stratified sampling to more comprehensively explore and compare the medication experience of patients in different regions. Second, this study adopted a cross-sectional design, which limits causal inference. Future studies should adopt methods such as structural equation modeling to systematically explore the mechanisms underlying the relationships between variables to provide clearer guidance for intervention pathways. Meanwhile, future studies may conduct sensitivity analyses to further verify the reliability of the core findings. Finally, due to the nature of self-reported measures, the results may be affected by the subjective judgment bias of participants.

## 6. Conclusions

In summary, the medication experience of older adults with MCCs in rural China was at a moderate level. Key factors associated with medication experience included sociodemographic characteristics (age, marriage status, source of income, medical insurance schemes) and disease-related factors (duration of medication use), as well as safe medication knowledge, perceived social support, self-efficacy for chronic disease management, and medication errors. In clinical practice, primary healthcare professionals should focus on older adults who are advanced in age, have limited financial resources, or have inadequate medical insurance coverage. Measures such as simplifying medication regimens and optimizing prescription cost-effectiveness may help reduce their medication burden. In addition, strengthening patient education on medication knowledge, enhancing family and community social support networks, and promoting self-efficacy are essential for developing patient-centered, individualized medication management strategies. Furthermore, health authorities should improve the drug reimbursement list and primary care pharmaceutical services in rural areas to provide policy support for enhancing medication safety and experience among older adults with MCCs.

## Figures and Tables

**Table 1:** Univariate analysis of medication experience among rural older adults with MCCs (N = 539).

Items	N (%)	Medication Experience Score (Mean ± SD)	t/F	*p*
**Gender**			2.504 ^a^	0.013
Male	273 (50.65%)	118.96 ± 17.75		
Female	266 (49.35%)	115.27 ± 16.43		
**Age**			2.718 ^a^	0.007
60~75 years old	397 (73.7%)	118.31 ± 17.61		
>75 years old	142 (26.3%)	113.73 ± 15.50		
**Educational level**			20.615 ^b^	<0.001
Primary or below	337 (62.52%)	113.04 ± 16.85		
Junior high school	140 (25.97%)	123.60 ± 15.51		
Senior high school	52 (9.65%)	122.80 ± 14.74		
University or above	10 (1.86%)	135.40 ± 17.30		
**Marriage status**			1.323 ^a^	0.095
Married	441 (81.82%)	117.73 ± 16.85		
Others	98(18.18%)	114.52 ± 18.58		
**Monthly household income per capita (yuan)**			15.503 ^b^	<0.001
<2000	216 (40.07%)	111.80 ± 16.65		
2000~2999	238 (44.16%)	118.35 ± 16.00		
3000~3999	62 (11.50%)	126.96 ± 15.42		
4000~4999	17 (3.15%)	124.00 ± 19.81		
≥5000	6 (1.11%)	140.50 ± 17.28		
**Sources of income**			13.673 ^b^	<0.001
Child support	212 (39.33%)	113.37 ± 16.37		
Retirement pension	63 (11.69%)	125.76 ± 16.74		
Minimum living allowance	49 (9.09%)	110.61 ± 15.50		
Others	215 (39.89%)	119.82 ± 17.06		
**Medical Insurance Schemes**			38.931 ^b^	<0.001
Urban Employee Medical Insurance	31 (5.75%)	131.67 ± 13.74		
Urban and Rural Residents’ Medical Insurance	459 (85.16%)	117.92 ± 15.67		
Others	49 (9.09%)	100.63 ± 20.82		
**Types of Illness**			0.504 ^a^	0.877
2	349 (64.7%)	117.22 ± 18.05		
3–5	190 (35.3%)	116.98 ± 15.55		
**Duration of illness (years)**			4.853 ^b^	0.008
1~	100 (18.6%)	119.40 ± 16.07		
3~	140 (26.0%)	119.90 ± 17.41		
>5	299 (55.5%)	115.09 ± 17.24		
**Number of Medications**			2.471 ^b^	0.085
<2	187 (34.70%)	118.28 ± 17.15		
3~5	259 (48.05%)	117.59 ± 16.81		
>5	93 (17.25%)	113.61 ± 18.05		
**Duration of Medication Use (years)**			1.469 ^b^	0.222
1~	99 (18.4%)	118.48 ± 17.73		
3~	134 (24.9%)	119.20 ± 16.06		
5~	143 (26.5%)	115.93 ± 18.00		

Note: ^a^ denotes t-value, ^b^ denotes F-value.

**Table 2:** Medication experience, safe medication knowledge, perceived social support, self-efficacy for chronic disease management, and medication errors scores in rural older adults with MCCs (N = 539).

Item	Score (Mean ± SD)	Scoring Rate
Total score of medication experience	117.14 ± 17.19	65.00%
Sensory experience	16.94 ± 3.10	72.00%
Emotional experience	20.78 ± 3.40	70.00%
Cognitive experience	28.49 ± 5.25	62.22%
Behavioral experience	33.81 ± 6.79	61.81%
Relational experience	17.27 ± 3.21	72.00%
Safe medication knowledge	11.99 ± 5.84	30.56%
Perceived social support	58.08 ± 10.37	71.43%
Self-efficacy for chronic disease management	5.97 ± 1.58	59.7%
Medication errors	27.97 ± 5.31	72.50%

Score rate: Score rate = (Actual score/Theoretical maximum score) × 100%.

**Table 3:** Dummy variable setting and independent variable assignment situation.

Variables	Assignment of Variables		
Gender	Male = 1, Female = 2		
Age	60~75 years old = 1, >75 years old = 2		
Educational level	Primary or below = 1, Junior high school = 2, Senior high school = 3, University or above = 4		
Marriage status	Married = 1, Others = 2		
Monthly household income per capita (yuan)	<2000 = 1, 2000~2999 = 2, 3000~3999= 3, 4000~4999 = 4, ≥5000 = 5		
Sources of income (ref.: Child support)	Retirement pension = 1	Minimum living allowance = 0	Others = 0
	Retirement pension = 0	Minimum living allowance = 1	Others = 0
	Retirement pension = 0	Minimum living allowance = 0	Others = 1
Medical Insurance Schemes (ref.: Urban and Rural Residents’ Medical Insurance)	Urban Employee Medical Insurance = 1		Others = 0
	Urban Employee Medical Insurance = 0		Others = 1
Types of Illness	2 types = 1, 3–5 types = 2		
Duration of illness (years)	1~ = 1, 3~ = 2, >5 = 3		
Number of Medications	<2 types = 1, 3~5 types = 2, >5 types = 3		
Duration of Medication Use(years)	1~ = 1, 3~ = 2, 5~ = 3		
Safe medication knowledge	Original value entry		
Perceived social support	Original value entry		
Self-efficacy for chronic disease management	Original value entry		
Medication errors	Original value entry		

**Table 4:** Hierarchical multiple regression analysis of medication experience among rural older adults with MCCs.

Model		B	Std. Error	β	t	*p*	95.0% Confidence Interval for B		Collinearity Statistics	
							Lower Bound	Upper Bound	Tolerance	VIF
1	(Constant)	130.397	3.620		36.017	<0.001	123.285	137.509		
	Gender	−3.563	1.467	−0.104	−2.430	0.015	−6.444	−0.682	0.999	1.001
	Age	−4.085	1.706	−0.104	−2.394	0.017	−7.473	−0.733	0.968	1.033
	Marriage status	−2.369	1.930	−0.053	−1.227	0.220	−6.160	1.422	0.969	1.032
2	(Constant)	105.760	4.655		22.717	<0.001	96.614	114.905		
	Gender	−2.135	1.399	−0.062	−1.526	0.128	−4.882	0.613	0.969	1.032
	Age	−0.870	1.669	−0.022	−0.522	0.602	−4.148	2.407	0.893	1.120
	Marriage status	1.080	1.879	0.024	0.575	0.566	−2.612	4.772	0.901	1.109
	Educational level	4.016	1.101	0.174	3.646	<0.001	1.852	6.180	0.704	1.419
	Monthly household income per capita (yuan)	3.886	0.966	0.190	4.022	<0.001	1.988	5.784	0.718	1.392
	Sources of income (ref.: Child support)									
	Retirement pension	3.804	2.640	0.071	1.441	0.150	−1.383	8.991	0.658	1.519
	Minimum living allowance	−3.945	2.552	−0.066	−1.546	0.123	−8.957	1.067	0.880	1.136
	Others	3.034	1.678	0.086	1.809	0.071	−0.261	6.330	0.702	1.425
3	(Constant)	107.251	4.471		23.986	<0.001	98.467	116.035		
	Gender	−2.106	1.341	−0.061	−1.570	0.117	−4.741	0.529	0.969	1.032
	Age	−1.115	1.601	−0.028	−0.696	0.486	−4.259	2.030	0.893	1.120
	Marriage status	2.237	1.811	0.050	1.236	0.217	−1.320	5.794	0.893	1.120
	Educational level	3.681	1.072	0.159	3.434	<0.001	1.575	5.786	0.684	1.462
	Monthly household income per capita (yuan)	3.585	0.928	0.175	3.865	<0.001	1.763	5.407	0.717	1.395
	Sources of income (ref.: Child support)									
	Retirement pension	0.514	2.942	0.010	0.175	0.861	−5.265	6.292	0.488	2.051
	Minimum living allowance	−2.706	2.456	−0.045	−1.102	0.271	−7.532	2.119	0.873	1.145
	Others	2.466	1.613	0.070	1.529	0.127	−0.702	5.634	0.699	1.431
	Medical Insurance Schemes (ref.: Urban and Rural Residents’ Medical Insurance)									
	Urban Employee Medical Insurance	6.395	3.731	0.087	1.714	0.087	−0.934	13.724	0.577	1.732
	Others	−15.553	2.340	−0.260	−6.645	<0.001	−20.151	−10.955	0.962	1.039
4	(Constant)	108.574	5.463		19.874	<0.001	97.842	119.306		
	Gender	−2.321	1.346	−0.068	−1.724	0.085	−4.965	0.324	0.961	1.041
	Age	−1.128	1.606	−0.029	−0.703	0.483	−4.284	2.027	0.886	1.129
	Marriage status	2.057	1.822	0.046	1.129	0.260	−1.523	5.636	0.881	1.135
	Educational level	3.583	1.074	0.155	3.337	<0.001	1.474	5.692	0.681	1.468
	Monthly household income per capita (yuan)	3.708	0.954	0.181	3.889	<0.001	1.835	5.582	0.678	1.476
	Sources of income (ref.: Child support)									
	Retirement pension	0.690	2.942	0.013	0.234	0.815	−5.090	6.470	0.487	2.053
	Minimum living allowance	−2.528	2.463	−0.042	−1.026	0.305	−7.367	2.312	0.868	1.152
	Others	2.358	1.633	0.067	1.444	0.149	−0.849	5.565	0.681	1.469
	Medical Insurance Schemes (ref.: Urban and Rural Residents’ Medical Insurance)									
	Urban Employee Medical Insurance	5.973	3.736	0.081	1.599	0.110	−1.366	13.312	0.575	1.738
	Others	−15.195	2.354	−0.254	−6.455	<0.001	−19.819	−10.570	0.950	1.052
	Types of Illness	1.892	1.573	0.053	1.203	0.229	−1.197	4.981	0.771	1.297
	Duration of illness (years)	−2.209	1.546	−0.100	−1.429	0.154	−5.247	0.828	0.301	3.318
	Number of Medications	−1.404	1.131	−0.057	−1.242	0.215	−3.625	0.817	0.696	1.437
	Duration of Medication Use (years)	1.634	1.126	0.104	1.451	0.147	−0.578	3.846	0.289	3.459
5	(Constant)	55.605	4.894		11.362	<0.001	45.991	65.218		
	Gender	−0.835	0.993	−0.024	−0.840	0.401	−2.786	1.117	0.937	1.067
	Age	−2.761	1.178	−0.070	−2.344	0.019	−5.075	−0.447	0.875	1.143
	Marriage status	4.170	1.338	0.094	3.117	0.002	1.542	6.798	0.868	1.152
	Educational level	0.463	0.810	0.020	0.571	0.568	−1.129	2.054	0.635	1.574
	Monthly household income per capita (yuan)	0.757	0.708	0.037	1.069	0.285	−0.634	2.149	0.652	1.534
	Sources of income (ref.: Child support)									
	Retirement pension	0.003	2.150	0.000	0.001	0.999	−4.221	4.227	0.484	2.064
	Minimum living allowance	1.508	1.814	0.025	0.832	0.406	−2.055	5.072	0.850	1.177
	Others	3.766	1.193	0.107	3.156	0.002	1.422	6.109	0.677	1.476
	Medical Insurance Schemes (ref.: Urban and Rural Residents’ Medical Insurance)									
	Urban Employee Medical Insurance	2.311	2.731	0.031	0.846	0.398	−3.054	7.677	0.572	1.749
	Others	−8.168	1.772	−0.137	−4.610	<0.001	−11.649	−4.687	0.891	1.122
	Types of Illness	0.743	1.148	0.021	0.648	0.518	−1.511	2.997	0.769	1.300
	Duration of illness (years)	−1.685	1.128	−0.076	−1.494	0.136	−3.900	0.531	0.301	3.323
	Number of Medications	0.423	0.830	0.017	0.510	0.610	−1.207	2.053	0.687	1.456
	Duration of Medication Use (years)	1.343	0.822	0.085	1.633	0.103	−0.272	2.958	0.288	3.473
	Safe medication knowledge	1.043	0.100	0.355	10.431	<0.001	0.847	1.240	0.677	1.477
	Perceived social support	0.479	0.056	0.289	8.490	<0.001	0.368	0.589	0.676	1.479
	Self-efficacy for chronic disease management	2.764	0.362	0.254	7.636	<0.001	2.053	3.474	0.706	1.417
6	(Constant)	45.129	5.121		8.813	<0.001	35.069	55.188		
	Gender	−1.233	0.969	−0.036	−1.273	0.204	−3.137	0.670	0.932	1.073
	Age	−3.533	1.154	−0.090	−3.061	0.002	−5.800	−1.266	0.862	1.160
	Marriage status	4.368	1.302	0.098	3.355	<0.001	1.810	6.925	0.867	1.153
	Educational level	0.512	0.788	0.022	0.650	0.516	−1.036	2.060	0.635	1.574
	Monthly household income per capita (yuan)	0.595	0.690	0.029	0.863	0.388	−0.759	1.950	0.651	1.536
	Sources of income (ref.: Child support)									
	Retirement pension	−0.517	2.093	−0.010	−0.247	0.805	−4.629	3.595	0.484	2.068
	Minimum living allowance	1.663	1.765	0.028	0.942	0.346	−1.804	5.129	0.850	1.177
	Others	4.624	1.171	0.132	3.950	<0.001	2.324	6.923	0.666	1.502
	Medical Insurance Schemes (ref.: Urban and Rural Residents’ Medical Insurance)									
	Urban Employee Medical Insurance	2.752	2.658	0.037	1.035	0.301	−2.470	7.973	0.571	1.751
	Others	−7.451	1.728	−0.125	−4.311	<0.001	−10.846	−4.056	0.886	1.129
	Types of Illness	0.002	1.124	0.000	0.002	0.999	−2.207	2.210	0.758	1.319
	Duration of illness (years)	−1.767	1.097	−0.080	−1.611	0.108	−3.922	0.388	0.301	3.323
	Number of Medications	0.144	0.808	0.006	0.178	0.859	−1.445	1.732	0.684	1.462
	Duration of Medication Use (years)	1.703	0.802	0.108	2.123	0.034	0.127	3.280	0.286	3.496
	Safe medication knowledge	0.856	0.103	0.291	8.315	<0.001	0.654	1.058	0.604	1.655
	Perceived social support	0.487	0.055	0.294	8.887	<0.001	0.380	0.595	0.676	1.480
	Self-efficacy for chronic disease management	2.450	0.356	0.226	6.873	<0.001	1.750	3.151	0.688	1.454
	Medication errors	0.571	0.103	0.177	5.547	<0.001	0.369	0.774	0.731	1.368

Note: R^2^ = 0.615, adjusted R^2^ = 0.602, F = 46.131, *p* < 0.001. Durbin-Watson = 1.787.

## Data Availability

The data presented in this study are available on request from the corresponding authors due to ethical and privacy restrictions.
